# Intraoperative Transfusion of Autologous Blood Protects from Acute Kidney Injury after Pediatric Congenital Heart Surgery

**DOI:** 10.31083/j.rcm2411331

**Published:** 2023-11-24

**Authors:** Yuhan Sun, Xian Zeng, Shanshan Shi, Zhuo Shi, Ting Huang, Yong Fan, Yuqing Feng, Xudong Lu, Huilong Duan, Xiangming Fan, Qiang Shu, Haomin Li

**Affiliations:** ^1^Clinical Data Center, Children's Hospital, Zhejiang University School of Medicine, National Clinical Research Center for Child Health, 310052 Hangzhou, Zhejiang, China; ^2^The College of Biomedical Engineering and Instrument Science, Zhejiang University, 310027 Hangzhou, Zhejiang, China; ^3^CICU, Children's Hospital, Zhejiang University School of Medicine, 310052 Hangzhou, Zhejiang, China; ^4^Cardiac Surgery,Children's Hospital, Zhejiang University School of Medicine, 310052 Hangzhou, Zhejiang, China; ^5^CPB/ECMO Children's Hospital, Zhejiang University School of Medicine, 310052 Hangzhou, Zhejiang, China

**Keywords:** acute kidney injury, causal inference, multi-factor analysis, pediatric cardiac surgery, treatment effect evaluation

## Abstract

**Background:**

Acute kidney injury (AKI) is a common complication after pediatric cardiac surgery. And autologous blood transfusion (ABT) is an important predictor of postoperative AKI. Unlike previous studies, which mainly focused on the correlation between ABT and AKI, the current study focuses heavily on the causal relationship between them, thus providing guidance for the treatment of patients during hospitalization to reduce the occurrence of AKI.

**Methods:**

A retrospective cohort of 3386 patients extracted from the Pediatric Intensive Care database was used for statistical analysis, multifactorial analysis, and causal inference. Characteristics that were correlated with ABT and AKI were categorized as confounders, instrumental variables, and effect modifiers, and were entered into the DoWhy causal inference model to determine causality. The calculated average treatment effect (ATE) was compared with the results of the multifactorial analysis.

**Results:**

The adjusted odds ratio (OR) for ABT volume was obtained by multifactorial analysis as 0.964. The DoWhy model refute test was able to indicate a causal relationship between ABT and AKI. Any ABT reduces AKI about 15.3%–18.8% by different estimation methods. The ATE regarding the amount of ABT was –0.0088, suggesting that every 1 mL/kg of ABT reduced the risk of AKI by 0.88%.

**Conclusions:**

Intraoperative transfusion of autologous blood can have a protective effect against postoperative AKI.

## 1. Introduction

Acute kidney injury (AKI) after pediatric cardiac surgery is very common, and the incidence is approximately 52% in infants [1], and 9.6–42% in children [2, 3]. AKI after surgery increases the length of hospital stay as well as the cost of treatment, and increases mortality [4, 5, 6, 7]. Even when patients survive AKI, they have an increased likelihood of developing long-term adverse outcomes such as chronic kidney disease and end-stage renal disease [8].

The pathophysiology of cardiac surgery-associated AKI is very complex and probably includes renal ischemia-reperfusion injury, inflammation, oxidative stress, hemolysis, and nephrotoxins [9]. A number of preoperative variables have been identified as risk factors of AKI in adult patients [10], and some sensitive biomarkers were also identified in pediatric patients [11], but there are few of these factors that can be changed to reduce the occurrence of AKI. Therefore, some changeable procedural variables, such as duration of cardiopulmonary bypass (CPB), hemodilution, low oxygen delivery, perioperative anemia, and blood transfusion, have been studied for their impact on AKI prevention [12, 13, 14, 15]. Numerous observational studies have shown that perioperative allogeneic blood transfusion and AKI after cardiac surgery are independently associated with each other [15, 16]. Cardiac surgery with CPB causes some degree of ischemia-reperfusion-related kidney injury thereby increasing the risk of AKI, whereas anemia and transfusion further aggravate kidney injury. Intraoperative allogeneic blood transfusion, therefore, should be minimized.

One option to reduce allogeneic blood delivery is intraoperative autologous blood transfusion (ABT), the retrograde autologous priming of the CPB circuit that is one of the ABT has been recommended by guidelines [17, 18]. In one of our previous studies we developed a real-time predictive model for the occurrence of AKI in children within 7 days after cardiac surgery [19]. We found that the more autologous blood delivery lowers the risk of AKI in almost all models that predict AKI at different time points and time windows, as shown in **Supplementary Figs. 1,2,3**. However, the prediction model only shows a correlation between ABT and AKI and does not indicate that the transfusion of autologous blood is the cause of reduced AKI. Previously, few studies have investigated the causal relationship between ABT and AKI, focusing instead only on their correlation. In the present study, we aimed to quantitatively evaluate the causal relationship between ABT and AKI quantitatively by using causal inference, and to provide evidence that can effectively support decisions during treatment to reduce the risk of postoperative AKI.

## 2. Materials and Methods

### 2.1 Study Design and Sample

**Fig. 1.**
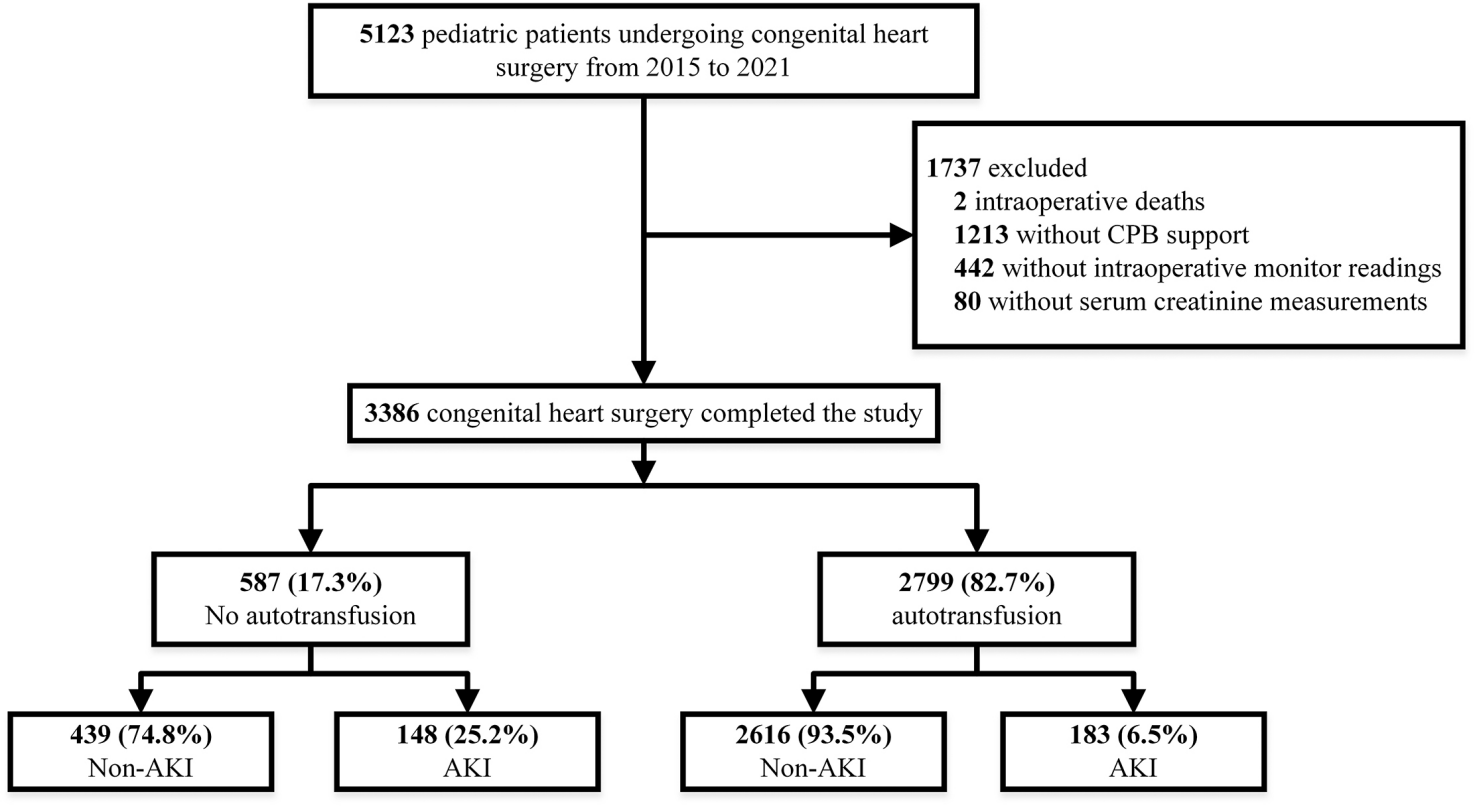
**Flowchart depicting the selection of patients**. After applying inclusion and exclusion criteria, we identified 2799 (82.7%) cases with autoblood transfusion and 587 (17.3%) cases without autoblood transfusion. The rate of postoperative AKI in the auto-blood group was 6.5%, while the rate of postoperative AKI in the non-auto-blood group was 25.2%. CPB, cardiopulmonary bypass; AKI, acute kidney injury.

The study was a retrospective cohort design that included pediatric patients (<18 years) who were hospitalized at least once for congenital heart surgery at the Children’s Hospital, Zhejiang University School of Medicine between December 2015 and June 2021. The following patients were not eligible for participation: (a) <2 serum creatinine (sCr) measurements at the time of hospitalization; (b) no CPB at the time of the surgery; (c) death during the surgery. Finally, a total of 3386 patients among 5123 participants were eligible for the study (Fig. 1). AKI was determined by either a 1.5-fold increase, or an absolute increase of 26.5 µmol/L, in SCr within 48 h after the guideline of KDIGO [20]. The sCr level measured before surgery was used as the reference value.

Institutional Review Board approval from the Children’s Hospital, Zhejiang University School of Medicine (2018-IRB-078) was obtained prior to the commencement of this study, and informed consent was waived because the research involved no more than minimal risk to patients. The waiver does not adversely affect the rights and welfare of the participants.

### 2.2 Transfusion Practice

During CPB supported pediatric heart surgery, the goal of transfusion is to maintain the hematocrit over 25%. In practice, for patients less than 10 kg, 1 unit of red blood cells is used in the priming fluid of CPB. For patients greater than 10 kg usually have a bloodless prime so long as the hematocrit >25% and hemoglbin >120 g/L. For severe cyanosis and severe pulmonary hypertension patients, perfusionists will add an additional 1 unit of red blood cells during surgery to keep the hematocrit at around 35%–40%. Autologous blood transfusion, especially for residual blood in the extracorporeal circulation machine, was used after surgery to achieve a postoperative hematocrit over 30% and in order to use as little donor blood as possbile.

### 2.3 Clinical Data

Demographic characteristics, intraoperative vital signs, and laboratory tests were collected from the electronic medical record system during the patient’s hospitalization. An overview of all 94 characteristics considered is prsented in **Supplementary Table 1**. They can be divided into static features (e.g., patient and procedure features) and time-series features (e.g., intraoperative vital signs, laboratory values, and blood gas analysis values). As the model we used in the causal analysis does not yet support dynamic data entry, we averaged the time-series values such as intraoperative variables and laboratory test variables. For categorical features such as type of surgery and surgical diagnosis, we used one-hot encoding to convert them to a binary representation. Also, because the patient cohort consisted of children, we changed the units of fresh frozen plasma (FFP) and ABT to mL/kg.

### 2.4 Statistical Analyses

In order to make preliminary relationship assumptions for the causal analysis that follows, statistical analyses of the variables in this study were done for the ABT and non-ABT groups and for the AKI and non-AKI groups. All normally distributed continuous variables are expressed as mean and standard deviation (SD) and compared using a two sample *t*-test. Skewed continuous data are expressed as median and interquartile range (IQR), and compared using the Kruskal-Wallis test. Categorical variables are expressed as count and percentage, and compared with Pearson’s Chi-squared test. Features between patients with and without AKI, and with and without ABT were all compared. Binary logistic regression analysis was used to calculate odds-ratios (OR) for the features that associated with the ABT. Unadjusted and adjusted multivariate logistic regressions were performed to evaluate the impact of volume of autologous blood on the AKI. The analyses were performed using Python package of Tableone and Statsmodels. All statistical tests were two-tailed, and *p* < 0.05 was considered statistically significant.

### 2.5 Model for Causal Inference

The fact that there is a link between ABT and AKI does not mean that there is also a causal relationship between them. The estimation of causal effects involves key assumptions about the data generation process, such as the directionality of the effect, the presence of instrumental variables or mediators, and whether all relevant confounding factors are observed [21]. Violation of any of these assumptions would lead to significant bias in the estimation of effects. However, unlike cross-validation of prediction models, there is no global validation method for causal estimation. Therefore, deriving different causal hypotheses and testing them as much as possible becomes the key to any analysis. In this study, we chose DoWhy [22], a Python library released by Microsoft for end-to-end causal inference, to study the causal relationship between intraoperative ABT and AKI after pediatric cardiac surgery. It allows for the explicit declaration of hypotheses through cause-and-effect diagrams and provides multiple validation tests to examine subsets of these hypotheses.

**Fig. 2.**
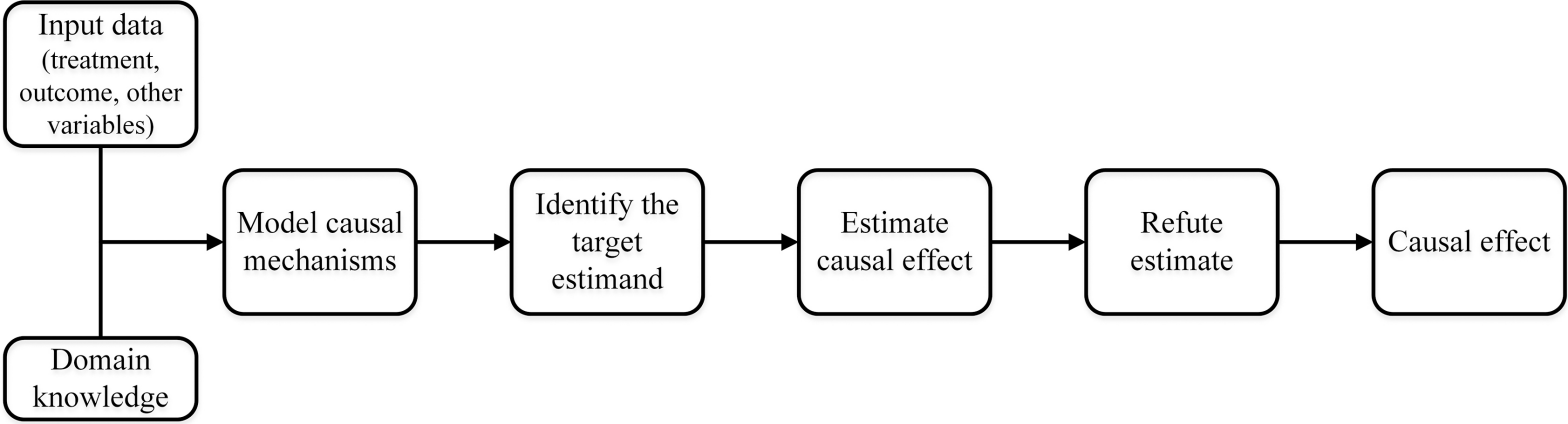
**The causal inference flowchart in DoWhy**. The four main steps of DoWhy causal inference: (1) Model: Construct a causal graph based on domain knowledge. (2) Identify: Formulate correct estimand based on the causal model. (3) Estimate: Use a suitable method to estimate effect. (4) Refute: Check robustness of estimate to assumption violations.

The entire causal inference process of DoWhy can be divided into four major steps (Fig. 2).

**Fig. 3.**
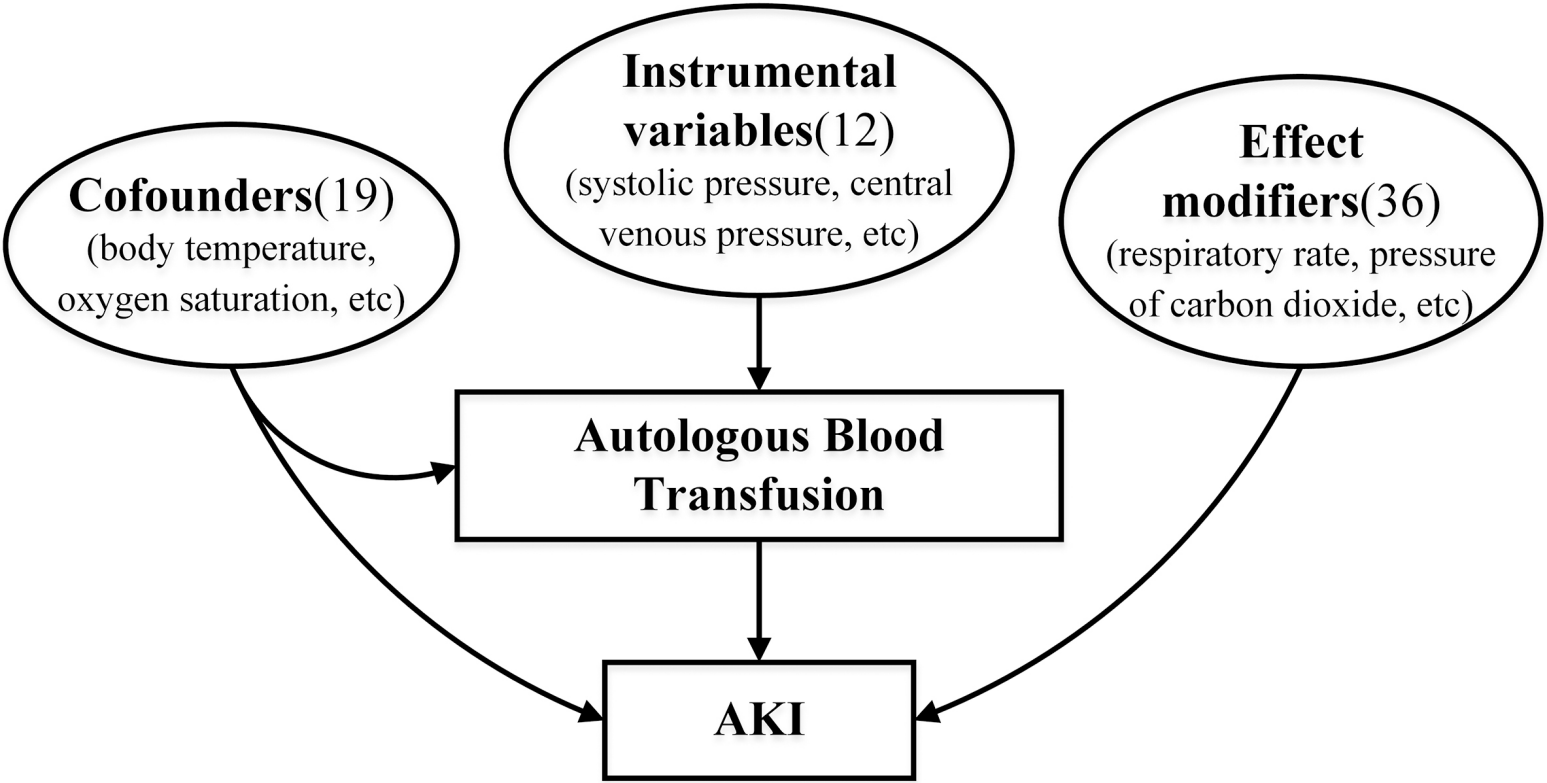
**The hypothetical causal model diagram**. According to statistical analysis, the characteristics can be divided into three categories: confounders, instrumental variables, effect modifiers. Confounders contain 19 features, including body temperature, oxygen saturation, *etc*. Instrumental variables contain 12 features, including systolic pressure, central venous pressure, *etc*. Effect modifiers contain 36 features, including respiratory rate, pressure of carbon dioxide, *etc*. AKI, acute kidney injury.

Firstly, ABT was defined as treatment and AKI was defined as outcome. Based on the above statistical analyses for patients in the ABT and non-ABT groups and in the AKI and non-AKI groups, characteristics significantly associated with autologous transfusion only were defined as instrumental variables (indirectly affecting the outcome by influencing the intervention), characteristics significantly associated with AKI only were defined as effect modifiers (directly affecting the outcome but not the intervention), and characteristics significantly associated with both were defined as confounders (affecting the intervention and outcome factors). The hypothetical model diagram was then generated based on three categories of these variables as shown in Fig. 3. The resulting hypothesized relationship diagrams do not need to be complete; DoWhy supports the automatic inclusion of the remaining variables as potential confounders. Then, DoWhy finds the expression that can identify the causal effect based on the constructed causal diagram and do-integral, and then use statistical methods to estimate the expression to calculate average treatment effect (ATE). After that, the following robustness checks were used to verify the correctness of the estimates:

Refute 1: Add a random confounder. Estimate whether the causal effect changes after adding a random variable as a confounder (expected result: No).

Refute 2: Placebo intervention. Estimate whether the causal effect changes when the real intervention variable is replaced by an independent random variable (expected result: the new causal effect goes to zero).

Refute 3: Data Subset Validation. Estimate whether the causal effect changes after replacing a given data set with a random subset (expected result: No).

## 3. Results

According to the inclusion and exclusion criteria, a total of 3386 patients were finally included in this study. The flow chart is shown in Fig. 1. **Supplementary Table 2** presents the statistical analysis of all characteristics of patients as to whether or not autologous blood was transfused. The statistically significant features in this analysis are presented in Table 1. Of the total sample, 2799 patients (82.7%) underwent intraoperative autotransfusion with a median and interquartile of 14.5 (10.2, 21.6) mL/kg for autotransfusion, whereas 587 patients were in the non-autotransfusion group. Significant differences (*p* < 0.05) were found between the ABT and non-ABT groups in characteristics such as systolic blood pressure, oxygen saturation, intraoperative transfusion of packed red blood cells (pRBC), and intraoperative transfusion of FFP. The risk of AKI was significant lower in the group with ABT (6.5%) than in the group without ABT (25.2%). **Supplementary Table 3** presents the statistical analysis of all characteristics of patients regarding with and without AKI. The statistically significant features in this analysis are presented in Table 2. Features such as age, weight, many of the lab tests, and intraoperative variables, were significantly different between the two groups. Based on the above analysis, these features were grouped into confounders, instrumental variables and effect modifiers (as shown in **Supplementary Table 4**). These variables were used to generated the causal model as shown in Fig. 3.

**Table 1.** **Statistically significant features compared between non-ABT and ABT groups**.

Features		Non-ABT	ABT	*p*-value
		n = 587	n = 2799	
Intraoperative vital signs				
	systolic pressure (mmHg)	81.5 [76.0, 90.3]	77.7 [76.0, 88.2]	0.022
	central venous pressure (cmH2O)	9.6 (6.2)	9.0 (5.0)	0.047
	body temperature (°C)	35.4 [34.8, 35.7]	35.4 [35.2, 35.7]	0.002
	heart rate (bpm)	135.6 [125.0, 147.4]	130.1 [125.0, 145.1]	<0.001
	pulse (bpm)	140.1 [127.0, 153.3]	133.9 [127.0, 150.5]	<0.001
	oxygen saturation (%)	98.7 [96.4, 99.2]	99.0 [97.6, 99.1]	0.004
Arterial blood gas values				
	methemoglobin (%)	1.0 [0.8, 1.2]	1.1 [0.9, 1.3]	<0.001
Preoperative Laboratory results				
	hematocrit (%)	36.2 [34.1, 38.9]	35.8 [33.5, 38.3]	0.003
	red blood cell count (${\mathrm{10}}^{12}$/L)	4.4 [4.1, 4.7]	4.4 [4.0, 4.7]	0.018
	hemoglobin (g/L)	120.2 [111.5, 128.4]	118.2 [109.2, 126.6]	0.002
	neutrophil (${\mathrm{10}}^{9}$/L)	34.9 [26.8, 46.2]	33.1 [25.6, 44.1]	0.017
	monocyte (${\mathrm{10}}^{9}$/L)	6.2 [4.9, 7.6]	6.3 [5.1, 7.9]	0.040
	basophilic granulocyte (${\mathrm{10}}^{9}$/L)	0.5 [0.3, 0.7]	0.4 [0.3, 0.6]	<0.001
	platelet distribution width (%)	13.0 [10.4, 15.7]	11.3 [9.6, 15.4]	<0.001
	mean platelet volume (fL)	9.5 (1.1)	9.6 (1.1)	0.011
	absolute basophil count (${\mathrm{10}}^{9}$/L)	0.0 [0.0, 0.1]	0.0 [0.0, 0.1]	<0.001
	high-sensitivity C-reactive protein (mg/L)	2.4 [1.0, 4.9]	1.9 [1.0, 3.8]	<0.001
Intraoperative variables				
	Mechanical ventilation time (hours)	17.0 [4.0, 25.0]	7.0 [4.0, 22.0]	<0.001
	pRBC transfusion during surgery (units)	1.0 [1.0, 2.0]	1.0 [1.0, 2.0]	0.010
	FFP transfusion during surgery (mL/kg)	21.4 [13.8, 43.1]	17.1 [12.2, 28.3]	<0.001
	ABT during surgery (mL/kg)	0.0 [0.0, 0.0]	14.5 [10.2, 21.6]	<0.001
PreSpO2 of right upper limb (%)		98.0 [96.0, 99.0]	98.0 [96.0, 99.0]	0.006
Other preoperative risk factors		76 (12.9)	493 (17.6)	0.007
Aristotle basic complexity score		6.0 [6.0, 6.8]	6.0 [5.0, 6.0]	0.003
STS mortality score		0.3 [0.2, 0.4]	0.2 [0.2, 0.4]	0.022
STS morbidity score		0.7 [0.5, 1.1]	0.7 [0.5, 1.1]	0.005
Number of major operations				0.037
	1	193 (32.9%)	1035 (37.0%)	
	>1	394 (67.1%)	1764 (63.0%)	
Number of defects				0.018
	1	181 (30.8%)	1008 (36.0%)	
	>1	406 (69.2%)	1791 (64.0%)	
Preoperative length of stay (days)		5.0 [3.0, 8.0]	4.0 [2.0, 7.0]	<0.001
Surgery procedure				
	Tricuspid valve plasty	34 (5.8%)	109 (3.9%)	0.049
	Mitral valve plasty	33 (5.6%)	104 (3.7%)	0.044
	PFO, primary closure	164 (27.9%)	635 (22.7%)	0.008
	ASD repair, patch	125 (21.3%)	810 (28.9%)	<0.001
Surgery diagnosis				
	PFO	168 (28.6%)	641 (22.9%)	0.004
	ASD, secundum	264 (45.0%)	1411 (50.4%)	0.019
AKI		148 (25.2%)	183 (6.5%)	<0.001

Data are presented as median (interquartile range) or mean (standard deviation) or number (%). pRBC, packed red blood cell; FFP, fresh frozen plasma; ABT, autologous blood transfusion; PreSpO2, preoperative oxygen saturation; STS, Society of Thoracic Surgeons; PFO, patent foramen ovale; ASD, atrial septal defect; AKI, acute kidney injury.

**Table 2.** **Statistically significant features compared between non-AKI and AKI groups**.

Features		Non-AKI	AKI	*p*-value
		n = 3055	n = 331	
Intraoperative vital signs				
	body temperature (°C)	35.4 [35.1, 35.7]	35.3 [34.8, 35.6]	<0.001
	respiratory rate (bpm)	24.0 [23.6, 26.2]	24.8 [23.1, 27.8]	0.008
	oxygen saturation (%)	99.0 [97.6, 99.2]	98.3 [95.6, 99.1]	<0.001
Arterial blood gas values				
	bicarbonate (mM)	22.5 (2.6)	22.9 (2.8)	0.048
	hemoglobin (g/L)	112.7 (18.6)	116.7 (24.1)	0.004
	pressure of oxygen (mmHg)	178.1 [137.8, 202.9]	166.8 [94.0, 200.6]	0.001
	hematocrit (%)	34.8 (5.6)	36.0 (7.3)	0.004
	oxygen saturation (%)	99.1 [98.3, 99.5]	98.9 [94.8, 99.5]	0.002
	methemoglobin (%)	1.1 [0.9, 1.3]	1.0 [0.8, 1.2]	<0.001
	calcium ${\mathrm{Ca}}^{2+}$ (mM)	1.2 (0.1)	1.2 (0.1)	0.002
	lactate	1.3 [0.9, 1.8]	1.4 [1.0, 2.2]	<0.001
Preoperative Laboratory results				
	hematocrit (%)	35.9 [33.6, 38.3]	36.2 [34.1, 39.4]	0.018
	platelet count (${\mathrm{10}}^{9}$/L)	322.2 (97.4)	306.5 (96.5)	0.005
	hemoglobin (g/L)	118.3 [109.5, 126.7]	120.0 [110.7, 129.6]	0.014
	mean corpuscular volume (fL)	83.3 (7.9)	85.4 (9.8)	<0.001
	mean corpuscular hemoglobin (pg)	27.4 (3.0)	28.1 (3.7)	0.001
	red blood cell distribution width (%)	13.4 [12.7, 14.5]	13.7 [12.7, 15.2]	0.004
	eosinophil (${\mathrm{10}}^{9}$/L)	2.2 [1.3, 3.5]	1.9 [1.1, 3.2]	0.008
	neutrophil (${\mathrm{10}}^{9}$/L)	33.2 [25.7, 43.7]	37.2 [27.4, 51.2]	<0.001
	lymphocyte (${\mathrm{10}}^{9}$/L)	56.4 [45.9, 64.4]	52.0 [39.2, 62.4]	<0.001
	basophilic granulocyte (${\mathrm{10}}^{9}$/L)	0.4 [0.3, 0.6]	0.4 [0.3, 0.7]	0.034
	platelet distribution width (%)	11.4 [9.7, 15.4]	13.5 [10.4, 15.7]	<0.001
	thrombocytocrit (%)	0.3 (0.1)	0.3 (0.1)	0.006
	absolute value of basophils (${\mathrm{10}}^{9}$/L)	0.0 [0.0, 0.1]	0.0 [0.0, 0.1]	0.022
	absolute lymphocyte count (${\mathrm{10}}^{9}$/L)	4.8 [3.6, 6.3]	4.6 [3.3, 5.9]	0.005
	absolute neutrophil count (${\mathrm{10}}^{9}$/L)	3.1 [2.3, 4.2]	3.4 [2.5, 5.0]	<0.001
	high-sensitivity C-reactive protein (mg/L)	1.9 [1.0, 3.9]	2.7 [1.3, 5.4]	<0.001
Demographics				
	Age (months)	12.6 [5.1, 30.6]	5.6 [1.9, 15.3]	<0.001
	Height (cm)	74.0 [63.0, 92.0]	64.0 [55.0, 78.0]	<0.001
	Weight (kg)	9.0 [6.0, 12.9]	6.3 [4.3, 9.6]	<0.001
Intraoperative/postoperatrive variables				
	Operation time (mins)	123.0 [107.0, 150.5]	148.0 [119.0, 207.0]	<0.001
	Emergency Operation	28 (0.9%)	17 (5.1%)	<0.001
	Cardiopulmonary bypass time (mins)	59.0 [47.0, 78.0]	85.0 [59.0, 133.5]	<0.001
	Cross clamp time (mins)	39.0 [28.0, 53.0]	53.0 [38.5, 91.0]	<0.001
	Mechanical ventilation time (hours)	6.0 [4.0, 21.0]	24.0 [16.0, 90.0]	<0.001
	pRBC transfusion during surgery (units)	1.0 [1.0, 1.5]	2.0 [1.0, 3.0]	<0.001
	FFP transfusion during surgery (mL/kg)	16.9 [12.2, 27.3]	39.7 [17.6, 81.1]	<0.001
	ABT during surgery (mL/kg)	12.6 [8.0, 19.3]	10.0 [0.0, 24.0]	0.001
PreSpO2 of right upper limb (%)		98.0 [97.0, 99.0]	97.0 [91.5, 98.0]	<0.001
PostSpO2 of right upper limb (%)		98.0 [97.0, 99.0]	98.0 [97.0, 99.0]	0.048
Previous congenital heart surgery		42 (1.4%)	12(3.6%)	0.003
Other preoperative risk factors		483 (15.8%)	86 (26.0%)	<0.001
Other malformation		176 (5.8%)	31 (9.4%)	0.013
RACHS-1 category				<0.001
	1	808 (26.4%)	42 (12.7%)	
	2	1843 (60.3%)	195 (58.9%)	
	3	353 (11.6%)	70 (21.1%)	
	4	51 (1.7%)	24 (7.3%)	
Aristotle basic complexity score		6.0 [4.0, 6.0]	6.0 [6.0, 8.7]	<0.001
STS mortality score		0.2 [0.2, 0.4]	0.4 [0.2, 0.7]	<0.001
STS morbidity score		0.7 [0.5, 1.1]	1.1 [0.6, 1.3]	<0.001
Number of major operations				<0.001
	1	1162 (38.0%)	66 (19.9%)	
	>1	1893 (62.0%)	265 (80.1%)	
Number of defects				<0.001
	1	1130 (37.0%)	59 (17.8%)	
	>1	1925 (63.0%)	272 (82.2%)	
Preoperative length of stay (days)		4.0 [2.0, 7.0]	7.0 [4.0, 11.0]	<0.001
Surgery procedure				
	TOF repair, ventriculotomy, transannular patch	87 (2.8%)	23 (6.9%)	<0.001
	Arterial switch operation	32 (1.0%)	18 (5.4%)	<0.001
	TAPVC repair	70 (2.3%)	19 (5.7%)	<0.001
	PDA closure	713 (23.3%)	140 (42.3%)	<0.001
	ASD repair, patch	867 (28.4%)	68 (20.5%)	0.003
	ASD repair, primary closure	645 (21.1%)	91 (27.5%)	0.009
Surgery diagnosis				
	TOF	127 (4.2%)	25 (7.6%)	0.007
	CoA	64 (2.1%)	28 (8.5%)	<0.001
	PDA	716 (23.4%)	140 (42.3%)	<0.001

Data are presented as median (interquartile range) or mean (standard deviation) or number (%). AKI, acute kidney injury; pRBC, packed red blood cell; FFP, fresh frozen plasma; ABT, autologous blood transfusion; PreSpO2, preoperative oxygen saturation; PostSpO2, postoperative oxygen saturation; RACHS-1, risk adjustment for congenital heart surgery; STS, society of thoracic surgeons; TOF, tetralogy of fallot; TAPVC, total anomalous pulmonary venous connection; PDA, patent ductus arteriosus; ASD, atrial septal defect; CoA, coarctation of the aorta.

**Fig. 4.**
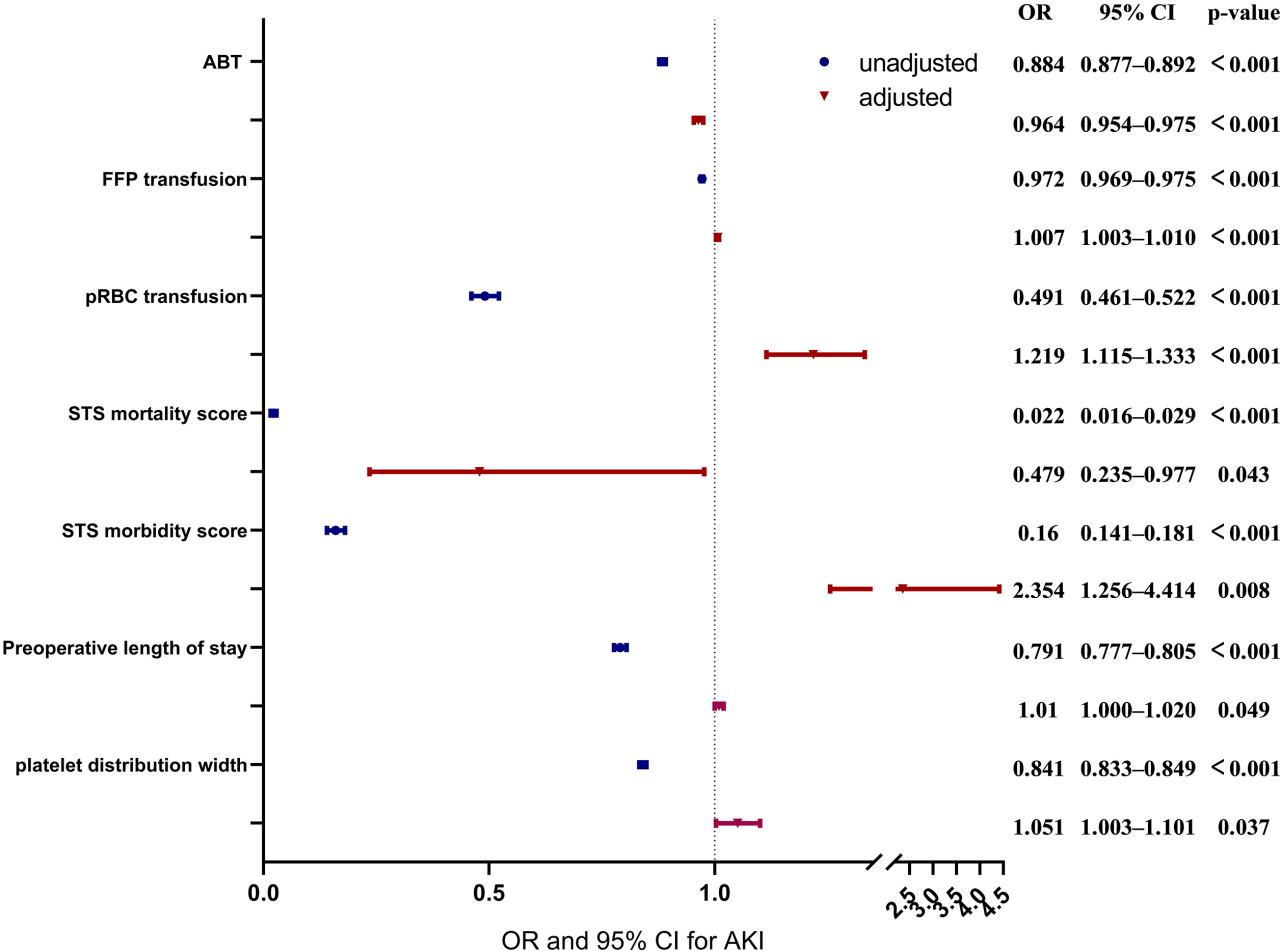
**Forest plots for the association between confounders and autologous blood transfusion (ABT), and aute kidney injury (AKI)**. This chart includes unadjusted and adjusted odds ratio (OR), 95% confidence intervals (CI) and *p* values of ABT, fresh frozen plasma (FFP) transfusion, packed red blood cell (pRBC) transfusion, Society of Thoracic Surgeons (STS) mortality score, STS morbidity score, preoperative length of stay and platelet distribution width.

The result of multiple logistic regression for ABT and confounders is presented in **Supplementary Table 5** and the significant associated features based on multifactorial logistic regression analysis are presented in Table 3. Only 7 features were significantly associated with AKI after adjustment by other factors. Multiple regression analyses of these variables showed that ABT during surgery (adjusted OR: 0.964, 95% confidence interval [CI]: 0.954–0.975; *p* < 0.001), pRBC transfusion during surgery (adjusted OR: 1.219, 95% CI: 1.115–1.333; *p* < 0.001), and FFP transfusion during surgery (adjusted OR: 1.007, 95% CI: 1.003–1.010; *p* < 0.001) were significantly associated with AKI. In the univariate analysis, the use of all blood products (pRBC, FFP, and autologous blood) was associated with a reduced risk of AKI. In the multifactorial analysis, the use of autologous blood was still associated with a reduced risk of AKI, but the delivery of FFP and pRBC was associated with an increased risk of AKI (Fig. 4).

**Table 3.** **Confounders of ABT and AKI based on multifactorial logistic regression analysis**.

Features		OR	95% CI	*p*-value	Adjusted OR	95% CI	*p*-value
Laboratory results							
	platelet distribution width (%)	0.841	[0.833, 0.849]	<0.001	1.051	[1.003, 1.101]	0.037
Intraop/postop variables							
	pRBC transfusion during surgery (unit)	0.491	[0.461, 0.522]	<0.001	1.219	[1.115, 1.333]	<0.001
	FFP transfusion during surgery (mL/kg)	0.972	[0.969, 0.975]	<0.001	1.007	[1.003, 1.010]	<0.001
	ABT during surgery (mL/kg)	0.884	[0.877, 0.892]	<0.001	0.964	[0.954, 0.975]	<0.001
STS mortality score		0.022	[0.016, 0.029]	<0.001	0.479	[0.235, 0.977]	0.043
STS morbidity score		0.160	[0.141, 0.181]	<0.001	2.354	[1.256, 4.414]	0.008
Preoperative length of stay (days)		0.791	[0.777, 0.805]	<0.001	1.010	[1.000, 1.020]	0.049

AKI, acute kidney injury; OR, odds ratio; pRBC, packed red blood cell; FFP, fresh frozen plasma; ABT, autologous blood transfusion; STS, Society of Thoracic Surgeons.

Table 4 shows the ATE estimated by the assumptions causal graph model in DoWhy. Since the data type of ABT volume was not applicable to the three estimation methods of propensity-based stratification, propensity scoring matching and inverse propensity weighting, only the linear regression method was used to evaluate the effect of intervention. In addition, due to the low proportion of patients with postoperative AKI in our data (9.8%), which did not qualify for the refutation method using placebo intervention, there were only two refutation results in the evaluation of whether autologous blood was transfused. In summary, the ATE was stable and passed different refutational scenarios. The ATE regarding the amount of autologous blood transfused was –0.0088, suggesting that every 1 mL/kg of ABT reduced the risk of AKI by 0.88%. Any ABT will reduce AKI by about 15.8%–18.8% by different estimation methods.

**Table 4.** **The average treatment effect (ATE) estimated by different methods and refutational scenarios**.

Estimation methods and refute method		ABT volume (mL/kg)	Any ABT (true/false)
Propensity-based stratification	ATE	/	–0.188
	Refute1 (*p*-value)	/	–0.180 (0.20)
	Refute2 (*p*-value)	/	/
	Refute3 (*p*-value)	/	–0.177 (0.21)
Propensity score matching	ATE	/	–0.160
	Refute1 (*p*-value)	/	–0.151 (0.28)
	Refute2 (*p*-value)	/	/
	Refute3 (*p*-value)	/	–0.153 (0.33)
Inverse propensity weighting	ATE	/	–0.153
	Refute1 (*p*-value)	/	–0.152 (0.29)
	Refute2 (*p*-value)	/	/
	Refute3 (*p*-value)	/	–0.152 (0.49)
Linear regression	ATE	–0.0088	–0.158
	Refute1 (*p*-value)	–0.0088 (0.49)	–0.158 (0.39)
	Refute2 (*p*-value)	0.000 (0.00)	0.000 (0.00)
	Refute3 (*p*-value)	–0.0087 (0.45)	–0.156 (0.47)

ABT, autologous blood transfusion.

## 4. Discussion

In the past, most research has been conducted using randomized controlled trial (RCT) experiments to compare differences between experimental and control groups to make causal inferences [23, 24]. Although there is less bias in the data and more control over data selection in RCT experiments than in retrospective studies, RCT experiments are usually expensive, time-consuming, and in many cases infeasible. Based on the relevance of the present study to the content of previous studies, we chose to conduct the experiment directly using existing retrospective data, taking into account the time period, expenditure costs and sample size of the data. For the present study, we used the results of DoWhy (https://github.com/py-why/dowhy) [22], a relatively new causal analysis framework in the field. This causal analysis framework can be used not only to assess possible causal effects, but also to provide a variety of refutation tools to verify the reliability of such causal effects. If it happens that the causal effect one is assessing is a confounding association, rather than a causal relationship due to a very large number of factors, problems can usually be identified through this refutation process. In the present study, we used three different refutation methods to verify the reliability of the results.

Similar to previous reports [25], our investigation demonstrated that ABT was associated with a reduction in intraoperative allogeneic blood transfusion. In practice, we have a policy that the hematocrit be kept above 25% through different transfusion and ABT will reduce the need for other blood transfusions. The ABT group had a longer operative time, shorter mechanical ventilation time, and lower amount of FFP transfused than did the non-ABT group. However, unlike in other studies, the difference in the amount of intraoperative pRBC transfusion between the ABT group and the non-ABT group was not observed in our results. When single-factor analysis was performed, the amount of ABT, pRBC transfusion, and FFP transfusion during surgery were all associated with the reduction of AKI, but when multifactor analysis with other confounder characteristics was added, only the amount of ABT was associated with the reduction of AKI. Theoretically, ABT reduces the exposure of clotting factors and platelets outside the circulatory system to the harmful effects of CPB. Platelet count and function are preserved regardless of the method of collection or the length of storage [26]. Centrifugation equipment and autologous transfusion systems are available to collect whole blood and separate it into red blood cell concentrates and autologous platelet-rich plasmapheresis (aPRP) fractions [27]. The collected erythrocytes are reperfused according to the hemoglobin concentration, whereas the aPRP fraction is reperfused after detachment from CPB and reversal of anticoagulation. In a previous study, Zhou *et al*. [28] hypothesized that the use of autologous blood could reduce postoperative lung injury and shorten the duration of mechanical ventilation, which is possibly related to the reduction of allogeneic blood transfusion.

In making causal inferences with the help of DoWhy, we used different evaluators in the ATE assessment phase and performed rebuttal tests for this. Refute 1 and 3 yielded new ATEs very close to the original values and Refute 2 yielded new ATEs close to 0. All the refutation results verified that our hypothesis, that ABT was one of the causes that reduce AKI, was correct. This has significant implications for reducing the occurrence of AKI by intervening in the amount of intraoperative ABT. In the multifactorial analysis we found an OR of 0.964 after correction of ABT, meaning that every 1 mL/kg of ABT reduced the risk of AKI by 3.6%. After causal extrapolation, the ATE suggested that every 1 mL/kg of ABT reduced the risk of AKI by 0.88%. This difference between the two also reinforces the difference between correlation and causation; correlation alone does not determine clinical decision making but requires further determination of causality between the two.

The present study provides solid evidence to encourage use of ABT during pediatric cardiac surgery. In addition, the shortage of blood supply is becoming a major issue worldwide due to the 2019 coronavirus disease (COVID-19) pandemic. The World Health Organization has also announced guidelines for protecting the blood supply during a COVID-19 pandemic. A large amount of donor blood is consumed during cardiac surgery; ABT not only significantly reduces allogeneic blood requirement in cardiac surgery, but also reduces the risk of AKI after surgery. However, since this study only used data from a single center, the validity of this conclusion in other centers still needs to be verified subsequently.

There are several limitations to our study. First, due to the database, the components of blood transfusion were not categorized, such as dividing the input autologous blood into hemoglobin and platelets, so the mechanism of the effect of intraoperative ABT on AKI could not be elucidated in detail. Second, due to the limitations of the model input, averaging was performed for time-series data and did not make good use of dynamic data. Third, there was about a 10:1 distribution of non-AKI to AKI cases in our data sample, so we were unable to obtain all the results of the refutation method when using the DoWhy model for causal inference. Last, the failure to describe the relationships between other characteristics when formulating model hypotheses could have had some impact on the final results.

## 5. Conclusions

The present study demonstrated that ABT is causally related to the occurrence of AKI and that every 1 mL/kg of ABT reduced the risk of AKI by 0.88%. Despite the limitations, the results of this study suggest that ABT is an effective transfusion strategy, especially during blood shortages due to COVID-19.

## Data Availability

Data can be obtained from the corresponding author on reasonable request.

## Abbreviations

ABT, autologous blood transfusion; AKI, aute kidney injury; ASD, atrial septal defect; ATE, average treatment effect; CoA, coarctation of the aorta; CPB, cardiopulmonary bypass; FFP, fresh frozen plasma; IQR, interquartile range; OR, odds ratio; PDA, patent ductus arteriosus; PFO, patent foramen ovale; pRBC, packed red blood cells; RACHS-1, risk adjustment for congenital heart surgery; SD, standard deviation; STS, society of thoracic surgeons; TAPVC, total anomalous pulmonary venous connection; TOF, tetralogy of fallot; VSD, ventricular septal defect.

## Supplementary Material

Supplementary material associated with this article can be found, in the online version, at https://doi.org/10.31083/j.rcm2411331.
