# Supplementary material for: Intraoperative Transfusion of Autologous Blood Protects from Acute Kidney Injury after Pediatric Congenital Heart Surgery
Source: Rev Cardiovasc Med. 2023 Nov 24;24(11):331. doi: 10.31083/j.rcm2411331 (PMC11272828; doi:10.31083/j.rcm2411331)
Supplement: Supplementary file 1 [file 2153-8174-24-11-331-s1.docx]

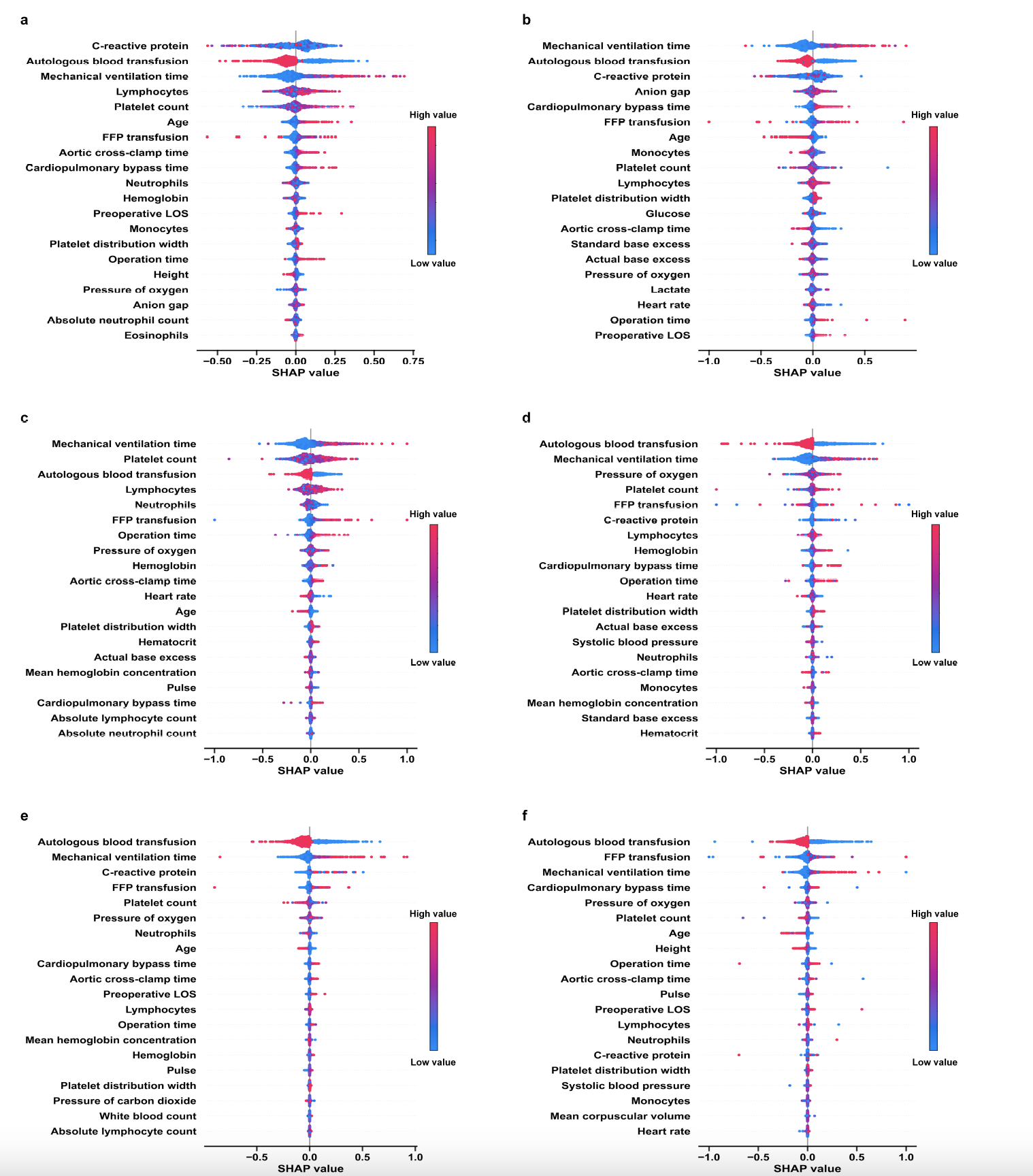


**Supplementary Fig. 1. The 15 features with highest mean absolute SHAP values in perspective 1 at different time point, quoted from “A time-aware attention model for prediction of acute kidney injury after pediatric cardiac surgery” [19].** a. onset-6h; b. onset-12h; c. onset-24h; d. onset-48h; e. onset-72h; f. onset-168h. On the y axis, the violin plot shows the full distribution of the SHAP values for each feature. The dot plot in the foreground shows a color coding of the actual value of the feature, resulting in the SHAP value as indicated on the x axis. The color coding is based on the percentile of the feature value with respect to the whole distribution.


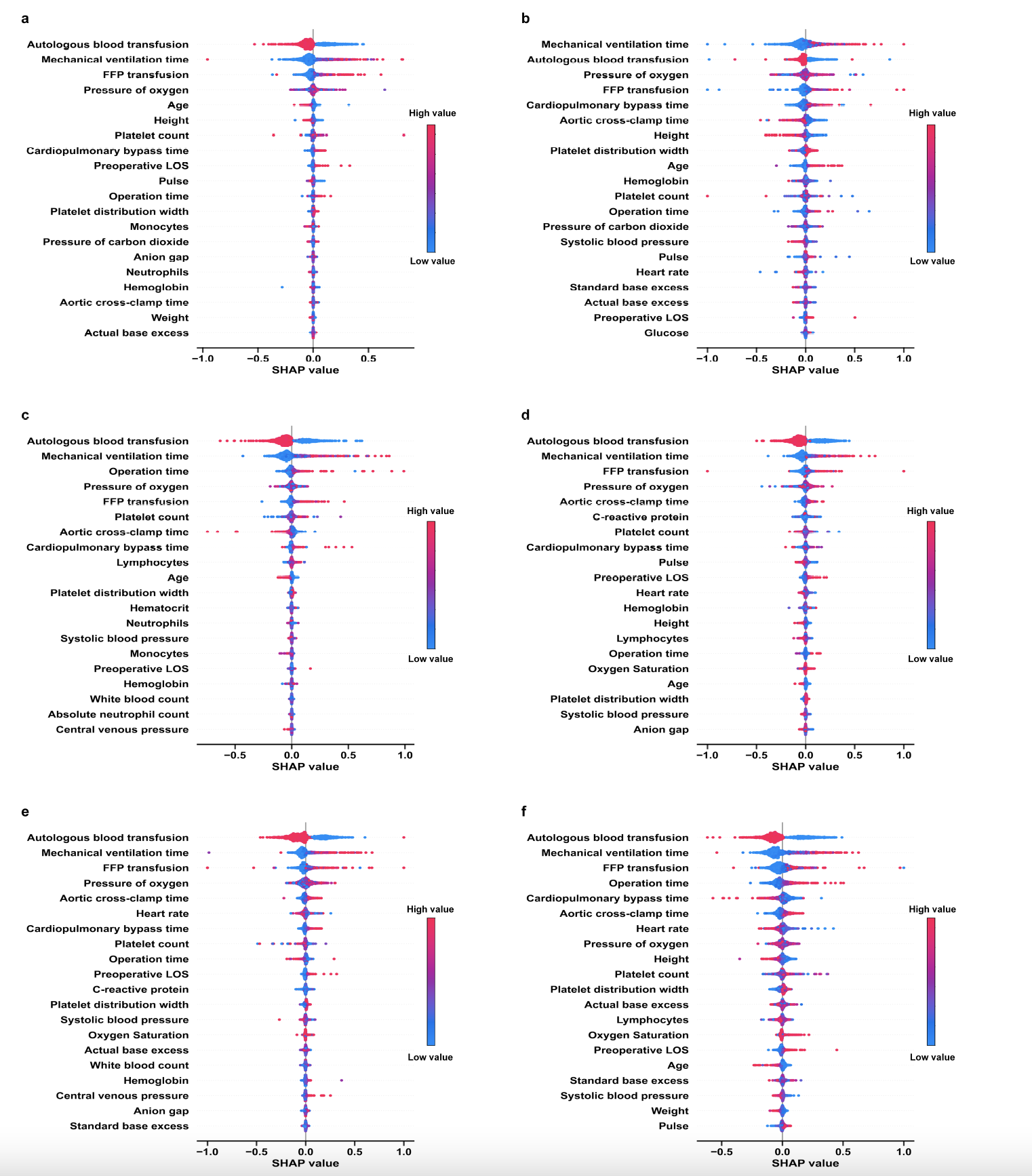


**Supplementary Fig. 2. The 15 features with highest mean absolute SHAP values in perspective 2 at different time point, quoted from “A time-aware attention model for prediction of acute kidney injury after pediatric cardiac surgery” [19].** a. surgery+0h; b. surgery+6h; c. surgery+12h; d. surgery+24h; e. surgery+48h; f. surgery+72h. On the y axis, the violin plot shows the full distribution of the SHAP values for each feature. The dot plot in the foreground shows a color coding of the actual value of the feature, resulting in the SHAP value as indicated on the x axis. The color coding is based on the percentile of the feature value with respect to the whole distribution.


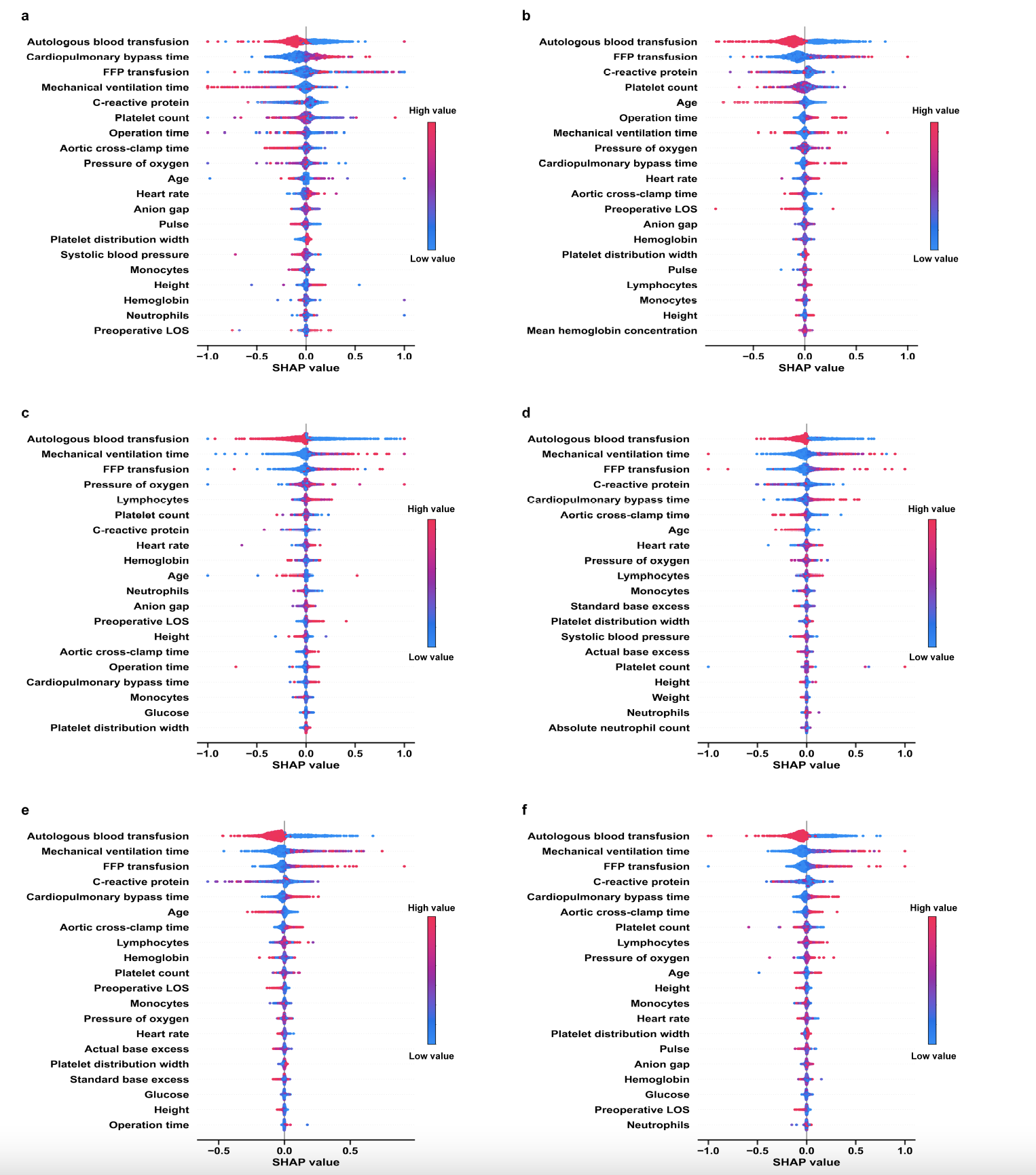


**Supplementary Fig. 3. The 15 features with highest mean absolute SHAP values in perspective 3 with different time window, quoted from “A time-aware attention model for prediction of acute kidney injury after pediatric cardiac surgery” [19].** a. random+6h; b. random+12h; c. random+24h; d. random+48h; e. random+72h; e. random+168h. On the y axis, the violin plot shows the full distribution of the SHAP values for each feature. The dot plot in the foreground shows a color coding of the actual value of the feature, resulting in the SHAP value as indicated on the x axis. The color coding is based on the percentile of the feature value with respect to the whole distribution.

**Supplementary Table 1**

| **Feature Group (no. features)** | **Features** |
| --- | --- |
| Patient characteristics (4) | Age, sex, height, weight |
| Preoperative conditions (5) | Preoperative length of stay, previous cardiac surgery, noncardiac malformation, other preoperative risk factors, preoperative oxygen saturation of right upper extremity |
| Intraoperative vital signs (8) | Systolic and diastolic arterial pressure, central venous pressure, heart rate, pulse, body temperature, oxygen saturation, respiratory rate |
| Arterial blood gas values (19) | Chloride, sodium, potassium, calcium, pH level, pressure of carbon dioxide, bicarbonate, standard base excess, hemoglobin, pressure of oxygen, hematocrit, carboxyhemoglobin, oxygen saturation, methemoglobin, standard bicarbonate, actual base excess, lactate, glucose, anion gap |
| Laboratory results (23) | Hematocrit, red blood count, platelet count, hemoglobin, white blood count, mean corpuscular volume, mean platelet volume, mean corpuscular hemoglobin, mean corpuscular hemoglobin concentration, red blood distribution width, platelet distribution width, eosinophils, absolute eosinophil count, neutrophils, absolute neutrophil count, lymphocytes, absolute lymphocyte count, monocytes, absolute monocyte count, basophils, absolute basophils count, plateletcrit, C-reactive protein |
| Surgery characteristics (35) | Elective, operation time, cardiopulmonary bypass time, aortic cross-clamp time, mechanical ventilation time, surgery diagnosis, surgery procedure, surgery risk scores including aristotle basic complexity score, RACHS-1 category, STS mortality score and STS morbidity score, number of operations, number of defects, pRBC transfusion during surgery, FFP transfusion during surgery, autologous blood transfusion during surgery, postoperative oxygen saturation of right upper limb |

The table lists all the variables used in our experiments, including patient characteristics, intraoperative vital signs, laboratory results and so on. RACHS-1: risk adjustment for congenital heart surgery, STS: society of thoracic surgeons, pRBC: packed red blood cells, FFP: fresh frozen plasma.

**Supplementary Table 2. Features compared between non- ABT and ABT groups**

| **features** | **all** | **Non-autotransfusion** | **autotransfusion** | **P-value** |
| --- | --- | --- | --- | --- |
| **Patient population** | 3386 | 587 | 2799 |  |
| **Intraoperative vital signs**  systolic pressure (mmHg)  diastolic pressure (mmHg)  central venous pressure (cmH2O)  body temperature (℃)  heart rate (bpm)  pulse (bpm)  respiratory rate (bpm)  oxygen saturation (%) | 78.4 [76.0,88.6]  47.3 (7.1)  9.1 (5.2)  35.4 [35.1,35.7]  131.5 [125.0,145.7]  135.2 [127.0,151.1]  24.0 [23.6,26.4]  99.0 [97.4,99.1] | 81.5 [76.0,90.3]  47.4 (5.7)  9.6 (6.2)  35.4 [34.8,35.7]  135.6 [125.0,147.4]  140.1 [127.0,153.3]  24.4 [23.1,27.0]  98.7 [96.4,99.2] | 77.7 [76.0,88.2]  47.3 (7.4)  9.0 (5.0)  35.4 [35.2,35.7]  130.1 [125.0,145.1]  133.9 [127.0,150.5]  24.0 [23.7,26.3]  99.0 [97.6,99.1] | 0.022  0.856  0.047  0.002  <0.001  <0.001  0.203  0.004 |
| **Arterial blood gas values**  chloridion Cl^-^ (mM)  sodion Na^+^ (mM)  potassium K^+^ (mM)  pH level  pressure of carbon dioxide (mmHg)  bicarbonate (mM)  standard base excess (mM)  hemoglobin (g/L)  pressure of oxygen (mmHg)  hematokrit (%)  carboxyhemoglobin (%)  oxygen saturation (%)  methemoglobin (%)  standard bicarbonate radical (mM)  calcium Ca^2+^ (mM)  actual base excess (mM)  lactate (mM)  glucose (mM)  anion gap (mM) | 108.7 (4.0)  136.8 (3.2)  3.8 (0.4)  7.4 (0.1)  36.3 [33.5,39.8]  22.6 (2.6)  -1.6 (2.7)  113.1 (19.3)  177.3 [134.5,202.5]  34.9 (5.8)  1.1 (0.4)  99.1 [98.2,99.5]  1.1 [0.9,1.3]  23.3 (2.2)  1.2 (0.1)  -1.4 (2.6)  1.3 [0.9,1.9]  5.8 [5.2,6.7]  7.9 (3.8) | 108.7 (3.9)  136.7 (3.2)  3.7 (0.4)  7.4 (0.1)  36.4 [33.5,39.7]  22.7 (2.5)  -1.5 (2.6)  114.2 (19.9)  174.9 [129.2,202.2]  35.2 (6.0)  1.1 (0.4)  99.1 [98.1,99.6]  1.0 [0.8,1.2]  23.3 (2.2)  1.2 (0.1)  -1.3 (2.6)  1.3 [0.9,1.9]  5.8 [5.2,6.7]  8.0 (4.0) | 108.7 (4.0)  136.9 (3.2)  3.8 (0.4)  7.4 (0.1)  36.3 [33.5,39.8]  22.5 (2.6)  -1.6 (2.7)  112.9 (19.2)  177.8 [135.9,202.8]  34.8 (5.8)  1.1 (0.4)  99.1 [98.2,99.5]  1.1 [0.9,1.3]  23.2 (2.2)  1.2 (0.1)  -1.4 (2.6)  1.3 [0.9,1.9]  5.8 [5.2,6.7]  7.9 (3.7) | 0.947  0.181  0.424  0.514  0.782  0.334  0.277  0.148  0.241  0.153  0.428  0.145  <0.001  0.319  0.199  0.320  0.941  0.861  0.382 |
| **Laboratory results**  Hematokrit (%)  red blood cell count (10^12/L)  platelet count (10^9/L)  hemoglobin (g/L)  white blood cell count (10^9/L)  mean corpuscular volume (fL)  mean hemoglobin concentration (g/L)  mean corpuscular hemoglobin (pg)  red blood cell distribution width (%)  eosinophil (10^9/L)  neutrophil (10^9/L)  lymphocyte (10^9/L)  monocyte (10^9/L)  basophilic granulocyte (10^9/L)  platelet distribution width (%)  mean platelet volume (fL)  thrombocytocrit (%)  absolute eosinophil count (10^9/L)  absolute basophil count (10^9/L)  absolute monocyte count (10^9/L)  absolute lymphocyte count (10^9/L)  absolute neutrophil count (10^9/L)  high-sensitivity C-reactive protein (mg/L) | 35.9 [33.7,38.4]  4.4 [4.1,4.7]  320.7 (97.4)  118.5 [109.6,126.9]  9.1 [7.5,11.2]  83.5 (8.1)  328.7 (13.4)  27.5 (3.1)  13.4 [12.7,14.6]  2.2 [1.3,3.5]  33.4 [25.7,44.4]  56.2 [45.2,64.3]  6.2 [5.0,7.8]  0.4 [0.3,0.6]  11.5 [9.7,15.4]  9.6 (1.1)  0.3 (0.1)  0.3 (0.2)  0.0 [0.0,0.1]  0.6 [0.4,0.8]  4.8 [3.5,6.2]  3.1 [2.3,4.3]  2.0 [1.0,4.0] | 36.2 [34.1,38.9]  4.4 [4.1,4.7]  317.3 (93.9  120.2 [111.5,128.4]  9.2 [7.6,11.3]  83.7 (8.4)  328.6 (14.1)  27.5 (3.2)  13.4 [12.7,14.7]  2.2 [1.2,3.6]  34.9 [26.8,46.2]  55.1 [44.0,63.8]  6.2 [4.9,7.6]  0.5 [0.3,0.7]  13.0 [10.4,15.7]  9.5 (1.1)  0.3 (0.1)  0.3 (0.2)  0.0 [0.0,0.1]  0.6 [0.4,0.7]  4.6 [3.5,6.1]  3.2 [2.4,4.4]  2.4 [1.0,4.9] | 35.8 [33.5,38.3]  4.4 [4.0,4.7]  321.4 (98.1)  118.2 [109.2,126.6]  9.1 [7.5,11.1]  83.5 (8.0)  328.7 (13.3)  27.5 (3.1)  13.4 [12.7,14.6]  2.2 [1.3,3.5]  33.1 [25.6,44.1]  56.4 [45.5,64.4]  6.3 [5.1,7.9]  0.4 [0.3,0.6]  11.3 [9.6,15.4]  9.6 (1.1)  0.3 (0.1)  0.3 (0.2)  0.0 [0.0,0.1]  0.6 [0.4,0.8]  4.8 [3.5,6.3]  3.1 [2.3,4.2]  1.9 [1.0,3.8] | 0.003  0.018  0.350  0.002  0.777  0.551  0.9  0.675  0.53  0.871  0.017  0.053  0.04  <0.001  <0.001  0.011  0.098  0.483  <0.001  0.057  0.317  0.052  <0.001 |
| **Demographics**  Age(months)  Gender  female  male  Height(cm)  Weight(kg) | 11.9 [4.5,28.9]  1714 (50.6%)  1672 (49.4%)  73.0 [62.0,91.0]  8.6 [5.8,12.5] | 10.0 [4.6,25.7]  293 (49.9%)  294 (50.1%)  71.0 [61.0,89.0]  8.3 [5.6,12.0] | 12.2 [4.5,29.7]  1421 (50.8%)  1378 (49.2%)  73.5 [62.0,92.0]  8.8 [5.9,12.5] | 0.167  0.741  0.100  0.191 |
| **Intraop/postop variables**  Operation time(mins)  Operation condition  selective operation  emergency operation  Cardiopulmonary bypass time(mins)  Cross clamp time (mins)  Mechanical ventilation time (hours)  pRBC transfusion during surgery (units)  FFP transfusion during surgery (ml/kg)  ABT during surgery (ml/kg) | 125.0 [108.0,156.0]  3341 (98.7%)  45 (1.3%)  60.0 [48.0,82.0]  40.0 [28.0,55.0]  7.0 [4.0,22.0]  1.0 [1.0,2.0]  17.6 [12.5,30.4]  12.5 [7.4,19.8] | 122.0 [106.0,156.0]  577 (98.3%)  10 (1.7%)  60.0 [48.0,83.0]  40.0 [28.5,55.0]  17.0 [4.0,25.0]  1.0 [1.0,2.0]  21.4 [13.8,43.1]  0.0 [0.0,0.0] | 126.0 [109.0,155.5]  2764 (98.7%)  35 (1.3%)  60.0 [48.0,82.0]  40.0 [28.0,55.0]  7.0 [4.0,22.0]  1.0 [1.0,2.0]  17.1 [12.2,28.3]  14.5 [10.2,21.6] | 0.212  0.501  0.811  0.911  <0.001  0.01  <0.001  <0.001 |
| **PreSpO2 of right upper limb (%)** | 98.0 [96.0,99.0] | 98.0 [96.0,99.0] | 98.0 [96.0,99.0] | 0.006 |
| **PostSpO2 of right upper limb (%)** | 98.0 [97.0,99.0] | 98.0 [97.0,99.0] | 98.0 [97.0,99.0] | 0.852 |
| **Number of previous congenital heart surgery**  0  1  2 | 3332 (98.4%)  51 (1.5%)  3 (0.1%) | 572 (97.4%)  15 (2.6%)  0 (0.0%) | 2760 (98.6%)  36 (1.3%)  3 (0.1%) | 0.053 |
| **Other preoperative risk factors**  none  have | 2817 (83.2)  569 (16.8) | 511 (87.1)  76 (12.9) | 2306 (82.4)  493 (17.6) | 0.007 |
| **Other malformation**  none  have | 3179 (93.9%)  207 (6.1%) | 561 (95.6%)  26 (4.4%) | 2618 (93.5%)  181 (6.5%) | 0.075 |
| **RACHS-1 category**  1  2  3  4 | 850 (25.1%)  2038 (60.2%)  423 (12.5%)  75 (2.2%) | 131 (22.3%)  352 (60.0%)  89 (15.2%)  15 (2.6%) | 719 (25.7%)  1686 (60.2%)  334 (11.9%)  60 (2.1%) | 0.085 |
| **Aristotle basic complexity score** | 6.0 [5.6,6.0] | 6.0 [6.0,6.8] | 6.0 [5.0,6.0] | 0.003 |
| **STS mortality score** | 0.2 [0.2,0.4] | 0.3 [0.2,0.4] | 0.2 [0.2,0.4] | 0.022 |
| **STS morbidity score** | 0.7 [0.5,1.1] | 0.7 [0.5,1.1] | 0.7 [0.5,1.1] | 0.005 |
| **Number of major operations**  1  2  3  4  5  6 | 1228 (36.3%)  1173 (34.6%)  754 (22.3%)  194 (5.7%)  33 (1.0%)  4 (0.1%) | 193 (32.9%)  218 (37.1%)  145 (24.7%)  24 (4.1%)  5 (0.9%)  2 (0.3%) | 1035 (37.0%)  955 (34.1%)  609 (21.8%)  170 (6.1%)  28 (1.0%)  2 (0.1%) | 0.037 |
| **Number of defects**  1  2  3  4  5  6  7 | 1189 (35.1%)  1091 (32.2%)  782 (23.1%)  253 (7.5%)  51 (1.5%)  19 (0.6%)  1 (0.0%) | 181 (30.8%)  186 (31.7%)  148 (25.2%)  54 (9.2%)  13 (2.2%)  4 (0.7%)  1 (0.2%) | 1008 (36.0%)  905 (32.3%)  634 (22.7%)  199 (7.1%)  38 (1.4%)  15 (0.5%)  0 (0.0%) | 0.018 |
| **Preoperative length of stay (days)** | 4.0 [2.0,7.0] | 5.0 [3.0,8.0] | 4.0 [2.0,7.0] | <0.001 |
| **Surgery procedure**  Tricuspid valve plasty  none  have  TOF repair, ventriculotomy, transannular patch  none  have  Arterial switch operation  none  have  Mitral valve plasty  none  have  TAPVC repair  none  have  PDA closure  none  have  PFO, primary closure  none  have  VSD repair, primary closure  none  have  VSD repair, patch  none  have  ASD repair, patch  none  have  ASD repair, primary closure  none  have  Partial atrioventricular passageway repair  none  have | 3243 (95.8%)  143 (4.2%)  3276 (96.8%)  110 (3.2%)  3336 (98.5%)  50 (1.5%)  3249 (96.0%)  137 (4.0%)  3297 (97.4%)  89 (2.6%)  2533 (74.8%)  853 (25.2%)  2587 (76.4%)  799 (23.6%)  3011 (88.9%)  375 (11.1%)  1856 (54.8%)  1530 (45.2%)  2451 (72.4%)  935 (27.6%)  2650 (78.3%)  736 (21.7%)  3326 (98.2%)  60 (1.8%) | 553 (94.2%)  34 (5.8%)  565 (96.3%)  22 (3.7%)  577 (98.3)  10 (1.7%)  554 (94.4%)  33 (5.6%)  571 (97.3%)  16 (2.7%)  440 (75.0%)  147 (25.0%)  423 (72.1%)  164 (27.9%)  524 (89.3%)  63 (10.7%)  310 (52.8%)  277 (47.2%)  462 (78.7%)  125 (21.3%)  455 (77.5%)  132 (22.5%)  576 (98.1%)  11 (1.9%) | 2690 (96.1%)  109 (3.9%)  2711 (96.9%)  88 (3.1%)  2759 (98.6)  40 (1.4%)  2695 (96.3%)  104 (3.7%)  2726 (97.4%)  73 (2.6%)  2093 (74.8%)  706 (25.2%)  2164 (77.3%)  635 (22.7%)  2487 (88.9%)  312 (11.1%)  1546 (55.2%)  1253 (44.8%)  1989 (71.1%)  810 (28.9%)  2195 (78.4%)  604 (21.6%)  2750 (98.2%)  49 (1.8%) | 0.049  0.534  0.754  0.044  0.984  0.969  0.008  0.827  0.304  <0.001  0.667  0.973 |
| **Surgery diagnosis**  TOF  none  have  CoA  none  have  PDA  none  have  PFO  none  have  VSD, type 1  none  have  VSD, type 2  none  have  ASD, primum  none  have  ASD, secundum  none  have | 3234 (95.5%)  152 (4.5%)  3294 (97.3%)  92 (2.7%)  2530 (74.7%)  856 (25.3%)  2577 (76.1%)  809 (23.9%)  2893 (85.4%)  493 (14.6%)  2021 (59.7%)  1365 (40.3%)  3328 (98.3%)  58 (1.7%)  1711 (50.5%)  1675 (49.5%) | 559 (95.2%)  28 (4.8%)  568 (96.8%)  19 (3.2%)  435 (74.1%)  152 (25.9%)  419 (71.4%)  168 (28.6%)  506 (86.2%)  81 (13.8%)  330 (56.2%)  257 (43.8%)  576 (98.1%)  11 (1.9%)  323 (55.0%)  264 (45.0%) | 2675 (95.6%)  124 (4.4%)  2726 (97.4%)  73 (2.6%)  2095 (74.8%)  704 (25.2%)  2158 (77.1%)  641 (22.9%)  2387 (85.3%)  412 (14.7%)  1691 (60.4%)  1108 (39.6%)  2752 (98.3%)  47 (1.7%)  1388 (49.6%)  1411 (50.4%) | 0.801  0.476  0.746  0.004  0.61  0.066  0.876  0.019 |
| AKI  none  have | 3055 (90.2)  331 (9.8) | 439 (74.8)  148 (25.2) | 2616 (93.5)  183 (6.5) | <0.001 |

Data are presented as median (interquartile range) or mean (standard deviation) or number (%). PreSpO2: preoperative oxygen saturation, PostSpO2: postoperative oxygen saturation, pRBC: packed red blood cell, FFP: fresh frozen plasma, ABT: autologous blood transfusion, RACHS-1: risk adjustment for congenital heart surgery, STS: society of thoracic surgeons, TOF: tetralogy of fallot, TAPVC: total anomalous pulmonary venous connection, PDA: patent ductus arteriosus, PFO: patent foramen ovale, VSD: ventricular septal defect, ASD: atrial septal defect, CoA: coarctation of the aorta. Chi-squared tests for the following variables may be invalid due to the low number of observations: Number of previous congenital heart surgery, RACHS-1 category, Number of major operations, Number of defects.

**Supplementary Table 3. Features compared between non- AKI and AKI groups**

| **features** | **all** | **Non-AKI** | **AKI** | **P-value** |
| --- | --- | --- | --- | --- |
| **Patient population** | 3386 | 3055 | 331 |  |
| **Intraoperative vital signs**  systolic pressure (mmHg)  diastolic pressure (mmHg)  central venous pressure (cmH2O)  body temperature (℃)  heart rate (bpm)  pulse (bpm)  respiratory rate (bpm)  oxygen saturation (%) | 78.4 [76.0,88.6]  47.3 (7.1)  9.1 (5.2)  35.4 [35.1,35.7]  131.5 [125.0,145.7]  135.2 [127.0,151.1]  24.0 [23.6,26.4]  99.0 [97.4,99.1] | 78.4 [76.0,88.7]  47.3 (7.2)  9.1 (5.2)  35.4 [35.1,35.7]  131.2 [125.0,145.6]  134.8 [127.0,150.7]  24.0 [23.6,26.2]  99.0 [97.6,99.2] | 78.3 [73.6,87.8]  47.3 (6.2)  9.5 (5.2)  35.3 [34.8,35.6]  134.6 [125.0,147.8]  138.3 [127.0,154.3]  24.8 [23.1,27.8]  98.3 [95.6,99.1] | 0.058  0.918  0.227  <0.001  0.147  0.137  0.008  <0.001 |
| **Arterial blood gas values**  chloridion Cl^-^ (mM)  sodion Na^+^ (mM)  potassium K^+^ (mM)  pH level  pressure of carbon dioxide (mmHg)  bicarbonate (mM)  standard base excess (mM)  hemoglobin (g/L)  pressure of oxygen (mmHg)  hematokrit (%)  carboxyhemoglobin (%)  oxygen saturation (%)  methemoglobin (%)  standard bicarbonate radical (mM)  calcium Ca^2+^ (mM)  actual base surplus (mM)  lactate (mM)  glucose (mM)  anion gap (mM) | 108.7 (4.0)  136.8 (3.2)  3.8 (0.4)  7.4 (0.1)  36.3 [33.5,39.8]  22.6 (2.6)  -1.6 (2.7)  113.1 (19.3)  177.3 [134.5,202.5]  34.9 (5.8)  1.1 (0.4)  99.1 [98.2,99.5]  1.1 [0.9,1.3]  23.3 (2.2)  1.2 (0.1)  -1.4 (2.6)  1.3 [0.9,1.9]  5.8 [5.2,6.7]  7.9 (3.8) | 108.7 (4.0)  136.8 (3.2)  3.8 (0.4)  7.4 (0.0)  36.3 [33.5,39.7]  22.5 (2.6)  -1.6 (2.6)  112.7 (18.6)  178.1 [137.8,202.9]  34.8 (5.6)  1.1 (0.4)  99.1 [98.3,99.5]  1.1 [0.9,1.3]  23.2 (2.2)  1.2 (0.1)  -1.4 (2.6)  1.3 [0.9,1.8]  5.8 [5.2,6.7]  7.9 (3.8) | 108.4 (4.1)  136.7 (3.3)  3.8 (0.4)  7.4 (0.1)  36.7 [33.9,41.0]  22.9 (2.8)  -1.3 (2.9)  116.7 (24.1)  166.8 [94.0,200.6]  36.0 (7.3)  1.1 (0.4)  98.9 [94.8,99.5]  1.0 [0.8,1.2]  23.4 (2.4)  1.2 (0.1)  -1.2 (2.9)  1.4 [1.0,2.2]  5.9 [5.2,6.8]  8.0 (3.9) | 0.191  0.474  0.168  0.355  0.069  0.048  0.13  0.004  0.001  0.004  0.58  0.002  <0.001  0.249  0.002  0.263  <0.001  0.136  0.591 |
| **Laboratory results**  hematokrit (%)  red blood cell count (10^12/L)  platelet count (10^9/L)  hemoglobin (g/L)  white blood cell count (10^9/L)  mean corpuscular volume (fL)  mean hemoglobin concentration (g/L)  mean corpuscular hemoglobin (pg)  red blood cell distribution width (%)  eosinophil (10^9/L)  neutrophil (10^9/L)  lymphocyte (10^9/L)  monocyte (10^9/L)  basophilic granulocyte (10^9/L)  platelet distribution width (%)  mean platelet volume (fL)  thrombocytocrit (%)  absolute eosinophil count (10^9/L)  absolute value of basophils (10^9/L)  absolute value of monocytes (10^9/L)  absolute lymphocyte count (10^9/L)  absolute neutrophil count (10^9/L)  high-sensitivity C-reactive protein (mg/L) | 35.9 [33.7,38.4]  4.4 [4.1,4.7]  320.7 (97.4)  118.5 [109.6,126.9]  9.1 [7.5,11.2]  83.5 (8.1)  328.7 (13.4)  27.5 (3.1)  13.4 [12.7,14.6]  2.2 [1.3,3.5]  33.4 [25.7,44.4]  56.2 [45.2,64.3]  6.2 [5.0,7.8]  0.4 [0.3,0.6]  11.5 [9.7,15.4]  9.6 (1.1)  0.3 (0.1)  0.3 (0.2)  0.0 [0.0,0.1]  0.6 [0.4,0.8]  4.8 [3.5,6.2]  3.1 [2.3,4.3]  2.0 [1.0,4.0] | 35.9 [33.6,38.3]  4.4 [4.1,4.7]  322.2 (97.4)  118.3 [109.5,126.7]  9.1 [7.5,11.1]  83.3 (7.9)  328.7 (13.2)  27.4 (3.0)  13.4 [12.7,14.5]  2.2 [1.3,3.5]  33.2 [25.7,43.7]  56.4 [45.9,64.4]  6.2 [5.0,7.8]  0.4 [0.3,0.6]  11.4 [9.7,15.4]  9.6 (1.1)  0.3 (0.1)  0.3 (0.2)  0.0 [0.0,0.1]  0.6 [0.4,0.8]  4.8 [3.6,6.3]  3.1 [2.3,4.2]  1.9 [1.0,3.9] | 36.2 [34.1,39.4]  4.4 [4.0,4.6]  306.5 (96.5)  120.0 [110.7,129.6]  9.3 [7.7,11.6]  85.4 (9.8)  328.3 (14.9)  28.1 (3.7)  13.7 [12.7,15.2]  1.9 [1.1,3.2]  37.2 [27.4,51.2]  52.0 [39.2,62.4]  6.4 [5.1,8.3]  0.4 [0.3,0.7]  13.5 [10.4,15.7]  9.7 (1.2)  0.3 (0.1)  0.2 (0.2)  0.0 [0.0,0.1]  0.6 [0.4,0.8]  4.6 [3.3,5.9]  3.4 [2.5,5.0]  2.7 [1.3,5.4] | 0.018  0.410  0.005  0.014  0.213  <0.001  0.597  0.001  0.004  0.008  <0.001  <0.001  0.092  0.034  <0.001  0.297  0.006  0.086  0.022  0.146  0.005  <0.001  <0.001 |
| **Demographics**  Age(months)  Gender  female  male  Height(cm)  Weight(kg) | 11.9 [4.5,28.9]  1714 (50.6%)  1672 (49.4%)  73.0 [62.0,91.0]  8.6 [5.8,12.5] | 12.6 [5.1,30.6]  1556 (50.9%)  1499 (49.1%)  74.0 [63.0,92.0]  9.0 [6.0,12.9] | 5.6 [1.9,15.3]  158 (47.7%)  173 (52.3%)  64.0 [55.0,78.0]  6.3 [4.3,9.6] | <0.001  0.295  <0.001  <0.001 |
| **Intraop/postop variables**  Operation time(mins)  Operation condition  selective operation  emergency operation  Cardiopulmonary bypass time(mins)  Cross clamp time (mins)  Mechanical ventilation time (hours)  pRBC transfusion during surgery (units)  FFP transfusion during surgery (ml/kg)  ABT during surgery (ml/kg) | 125.0 [108.0,156.0]  3341 (98.7%)  45 (1.3%)  60.0 [48.0,82.0]  40.0 [28.0,55.0]  7.0 [4.0,22.0]  1.0 [1.0,2.0]  17.6 [12.5,30.4]  12.5 [7.4,19.8] | 123.0 [107.0,150.5]  3027 (99.1%)  28 (0.9%)  59.0 [47.0,78.0]  39.0 [28.0,53.0]  6.0 [4.0,21.0]  1.0 [1.0,1.5]  16.9 [12.2,27.3]  12.6 [8.0,19.3] | 148.0 [119.0,207.0]  314 (94.9%)  17 (5.1%)  85.0 [59.0,133.5]  53.0 [38.5,91.0]  24.0 [16.0,90.0]  2.0 [1.0,3.0]  39.7 [17.6,81.1]  10.0 [0.0,24.0] | <0.001  <0.001  <0.001  <0.001  <0.001  <0.001  <0.001  0.001 |
| **PreSpO2 of right upper limb** (%) | 98.0 [96.0,99.0] | 98.0 [97.0,99.0] | 97.0 [91.5,98.0] | <0.001 |
| **PostSpO2 of right upper limb** (%) | 98.0 [97.0,99.0] | 98.0 [97.0,99.0] | 98.0 [97.0,99.0] | 0.048 |
| **Number of previous congenital heart surgery**  0  1  2 | 3332 (98.4%)  51 (1.5%)  3 (0.1%) | 3013 (98.6%)  39 (1.3%)  3 (0.1%) | 319 (96.4%)  12 (3.6%)  0(0.0%) | 0.003 |
| **Other preoperative risk factors**  none  have | 2817 (83.2%)  569 (16.8%) | 2572 (84.2%)  483 (15.8%) | 245 (74.0%)  86 (26.0%) | <0.001 |
| **Other malformation**  none  have | 3179 (93.9%)  207 (6.1%) | 2879 (94.2%)  176 (5.8%) | 300 (90.6%)  31 (9.4%) | 0.013 |
| **RACHS-1 category**  1  2  3  4 | 850 (25.1%)  2038 (60.2%)  423 (12.5%)  75 (2.2%) | 808 (26.4%)  1843 (60.3%)  353 (11.6%)  51 (1.7%) | 42 (12.7%)  195 (58.9%)  70 (21.1%)  24 (7.3%) | <0.001 |
| **Aristotle basic complexity score** | 6.0 [5.6,6.0] | 6.0 [4.0,6.0] | 6.0 [6.0,8.7] | <0.001 |
| **STS mortality score** | 0.2 [0.2,0.4] | 0.2 [0.2,0.4] | 0.4 [0.2,0.7] | <0.001 |
| **STS morbidity score** | 0.7 [0.5,1.1] | 0.7 [0.5,1.1] | 1.1 [0.6,1.3] | <0.001 |
| **Number of major operations**  1  2  3  4  5  6 | 1228 (36.3%)  1173 (34.6%)  754 (22.3%)  194 (5.7%)  33 (1.0%)  4 (0.1%) | 1162 (38.0%)  1069 (35.0%)  632 (20.7%)  167 (5.5%)  24 (0.8%)  1 (0.0%) | 66 (19.9%)  104 (31.4%)  122 (36.9%)  27 (8.2%)  9 (2.7%)  3 (0.9%) | <0.001 |
| **Number of defects**  1  2  3  4  5  6  7 | 1189 (35.1%)  1091 (32.2%)  782 (23.1%)  253 (7.5%)  51 (1.5%)  19 (0.6%)  1 (0.0%) | 1130 (37.0%)  1001 (32.8%)  656 (21.5%)  215 (7.0%)  39 (1.3%)  14 (0.5%)  0 (0.0%) | 59 (17.8%)  90 (27.2%)  126 (38.1%)  38 (11.5%)  12 (3.6%)  5 (1.5%)  1 (0.3%) | <0.001 |
| **Preoperative length of stay (days)** | 4.0 [2.0,7.0] | 4.0 [2.0,7.0] | 7.0 [4.0,11.0] | <0.001 |
| **Surgery procedure**  Tricuspid valve plasty  none  have  TOF repair, ventriculotomy, transannular patch  none  have  Arterial switch operation  none  have  Mitral valve plasty  none  have  TAPVC repair  none  have  PDA closure  none  have  PFO, primary closure  none  have  VSD repair, primary closure  none  have  VSD repair, patch  none  have  ASD repair, patch  none  have  ASD repair, primary closure  none  have  Partial atrioventricular passageway repair  none  have | 3243 (95.8%)  143 (4.2%)  3276 (96.8%)  110 (3.2%)  3336 (98.5%)  50 (1.5%)  3249 (96.0%)  137 (4.0%)  3297 (97.4%)  89 (2.6%)  2533 (74.8%)  853 (25.2%)  2587 (76.4%)  799 (23.6%)  3011 (88.9%)  375 (11.1%)  1856 (54.8%)  1530 (45.2%)  2451 (72.4%)  935 (27.6%)  2650 (78.3%)  736 (21.7%)  3326 (98.2%)  60 (1.8%) | 2930 (95.9%)  125 (4.1%)  2968 (97.2%)  87 (2.8%)  3023 (99.0%)  32 (1.0%)  2936 (96.1%)  119 (3.9%)  2985 (97.7%)  70 (2.3%)  2342 (76.7%)  713 (23.3%)  2336 (76.5%)  719 (23.5%)  2708 (88.6%)  347 (11.4%)  1660 (54.3%)  1395 (45.7%)  2188 (71.6%)  867 (28.4%)  2410 (78.9%)  645 (21.1%)  3001 (98.2%)  54 (1.8%) | 313 (94.6%)  18 (5.4%)  308 (93.1%)  23 (6.9%)  313 (94.6%)  18 (5.4%)  313 (94.6%)  18 (5.4%)  312 (94.3%)  19 (5.7%)  191 (57.7%)  140 (42.3%)  251 (75.8%)  80 (24.2%)  303 (91.5%)  28 (8.5%)  196 (59.2%)  135 (40.8%)  263 (79.5%)  68 (20.5%)  240 (72.5%)  91 (27.5%)  325 (98.2%)  6 (1.8%) | 0.311  <0.001  <0.001  0.228  <0.001  <0.001  0.849  0.132  0.102  0.003  0.009  0.873 |
| **Surgery diagnosis**  TOF  none  have  CoA  none  have  PDA  none  have  PFO  none  have  VSD, type 1  none  have  VSD, type 2  none  have  ASD, primum  none  have  ASD, secundum  none  have | 3234 (95.5%)  152 (4.5%)  3294 (97.3%)  92 (2.7%)  2530 (74.7%)  856 (25.3%)  2577 (76.1%)  809 (23.9%)  2893 (85.4%)  493 (14.6%)  2021 (59.7%)  1365 (40.3%)  3328 (98.3%)  58 (1.7%)  1711 (50.5%)  1675 (49.5%) | 2928 (95.8%)  127 (4.2%)  2991 (97.9%)  64 (2.1%)  2339 (76.6%)  716 (23.4%)  2329 (76.2%)  726 (23.8%)  2605 (85.3%)  450 (14.7%)  1810 (59.2%)  1245 (40.8%)  3002 (98.3%)  53 (1.7%)  1544 (50.5%)  1511 (49.5%) | 306 (92.4%)  25 (7.6%)  303 (91.5%)  28 (8.5%)  191 (57.7%)  140 (42.3%)  248 (74.9%)  83 (25.1%)  288 (87.0%)  43 (13.0%)  211 (63.7%)  120 (36.3%)  326 (98.5%)  5 (1.5%)  167 (50.5%)  164 (49.5%) | 0.007  <0.001  <0.001  0.643  0.441  0.127  0.94  0.978 |

Data are presented as median (interquartile range) or mean (standard deviation) or number (%). PreSpO2: preoperative oxygen saturation, PostSpO2: postoperative oxygen saturation, pRBC: packed red blood cell, FFP: fresh frozen plasma, ABT, autologous blood transfusion, RACHS-1: risk adjustment for congenital heart surgery, STS: society of thoracic surgeons, TOF: tetralogy of fallot, TAPVC: total anomalous pulmonary venous connection, PDA: patent ductus arteriosus, PFO: patent foramen ovale, VSD: ventricular septal defect, ASD: atrial septal defect, CoA: coarctation of the aorta. Chi-squared tests for the following variables may be invalid due to the low number of observations: Number of previous congenital heart surgery, RACHS-1 category, Number of major operations, Number of defects.

**Supplementary Table 4.** Classification of features

| **Feature Group (no. features)** | **Features** |
| --- | --- |
| Confounders (20) | body temperature, oxygen saturation, methemoglobin, hematocrit, hemoglobin, neutrophil, basophilic granulocyte, platelet distribution width, absolute value of basophils, high-sensitivity C-reactive protein, PreSpO2 of right upper limb, Mechanical ventilation time, pRBC transfusion during surgery, FFP transfusion during surgery, Other preoperative risk factors, Aristotle basic complexity score, STS mortality score, STS morbidity score, ASD Repair and Patch, Preoperative length of stay |
| instrumental variables (12) | Systolic pressure, central venous pressure, heart rate, pulse, red blood cell count, monocyte, mean platelet volume, Tricuspid Valve Plasty, Mitral Valve Plasty, PFO and Primary Closure, PFO, ASD Secundum |
| effect modifiers (35) | Respiratory rate, bicarbonate, hemoglobin, pressure of oxygen, hematocrit, oxygen saturation, calcium Ca^2+^, lactate, platelet count, mean corpuscular volume, mean corpuscular hemoglobin, red blood cell distribution width, eosinophil, lymphocyte, thrombocytocrit, absolute lymphocyte count, absolute neutrophil count, Age, Height, Weight, PostSpO2 of right upper limb, Operation time, Operation condition, Cardiopulmonary bypass time, Cross clamp time, RACHS-1 category, Non-cardiac Malformation, TOF Repair Ventriculotomy and Transannular Patch, Arterial Switch Operation, TAPVC Repair, PDA Closure, ASD Repair and PrimaryClosure, TOF, CoA, PDA |

This table lists the input features of the DoWhy model, divided into three categories: confounders, instrumental variables, and effect modifiers. Confounders mean characteristics associated with both autologous blood transfusion and AKI. Instrumental variables mean features associated with autologous blood transfusion only. effect modifiers mean features that are closely associated with AKI only. PreSpO2: preoperative oxygen saturation, PostSpO2: postoperative oxygen saturation, pRBC: packed red blood cell, FFP: fresh frozen plasma, RACHS-1: risk adjustment for congenital heart surgery, STS: society of thoracic surgeons, TOF: tetralogy of fallot, TAPVC: total anomalous pulmonary venous connection, PDA: patent ductus arteriosus, PFO: patent foramen ovale, VSD: ventricular septal defect, ASD: atrial septal defect, CoA: coarctation of the aorta. Chi-squared tests for the following variables may be invalid due to the low number of observations: Number of previous congenital heart surgery, RACHS-1 category, Number of major operations, Number of defects.

**Supplementary Table 5. Univariate and multifactorial logistic regression analysis of ABT and confounders.**

| **features** | **OR** | **95%CI** | **P-value** | **Adjusted OR** | **95%CI** | **P-value** |
| --- | --- | --- | --- | --- | --- | --- |
| **Intraoperative vital signs**  body temperature (℃)  oxygen saturation (%) | 0.938  0.977 | [0.935,0.942]  [0.976,0.978] | <0.001  <0.001 | 0.989  0.989 | [0.928,1.053]  [0.967,1.012] | 0.724  0.342 |
| **Arterial blood gas values**  Methemoglobin (%) | 0.123 | [0.110,0.138] | <0.001 | 0.774 | [0.489,1.223] | 0.272 |
| **Laboratory results**  Hematocrit (%)  hemoglobin (g/L)  neutrophil (10^9/L)  basophilic granulocyte (10^9/L)  platelet distribution width (%)  absolute value of basophils (10^9/L)  high-sensitivity C-reactive protein (mg/L) | 0.942  0.982  0.945  0.014  0.841  0.000  0.674 | [0.939,0.944]  [0.981,0.983]  [0.942,0.948]  [0.010,0.018]  [0.833,0.849]  [0.000,0.000]  [0.652,0.696] | <0.001  <0.001  <0.001  <0.001  <0.001  <0.001  <0.001 | 1.007  0.990  1.001  1.345  1.051  2.228  1.003 | [0.936,1.082]  [0.968,1.013]  [0.992,1.010]  [0.716,2.525]  [1.003,1.101]  [0.006,776.416]  [0.987,1.020] | 0.858  0.404  0.829  0.356  **0.037**  0.789  0.693 |
| **PreSpO2 of right upper limb** | 0.977 | [0.976,0.978] | <0.001 | 0.985 | [0.968,1.002] | 0.076 |
| **Intraop/postop variables**  Mechanical ventilation time (mins)  pRBC transfusion during surgery (units)  FFP transfusion during surgery (mL/kg)  ABT during surgery (mL/kg) | 0.992  0.491  0.9720.884 | [0.990,0.994]  [0.461,0.522]  [0.969,0.975]  [0.877,0.892] | <0.001  <0.001  <0.001  <0.001 | 1.003  1.219  1.007  0.964 | [0.999,1.006]  [1.115,1.333]  [1.003,1.010]  [0.954,0.975] | 0.108  **<0.001**  **<0.001**  **<0.001** |
| **Aristotle basic complexity score** | 0.715 | [0.702,0.728] | <0.001 | 1.053 | [0.938,1.182] | 0.384 |
| **STS mortality score** | 0.022 | [0.016,0.029] | <0.001 | 0.479 | [0.235,0.977] | **0.043** |
| **STS morbidity score** | 0.160 | [0.141,0.181] | <0.001 | 2.354 | [1.256,4.414] | **0.008** |
| **Preoperative length of stay** (days) | 0.791 | [0.777,0.805] | <0.001 | 1.010 | [1.000,1.020] | **0.049** |
| **Other preoperative risk factors** | 0.178 | [0.142,0.224] | <0.001 | 0.825 | [0.577,1.178] | 0.290 |
| **Surgery procedure**  ASD repair, patch | 0.078 | [0.061,0.100] | <0.001 | 0.776 | [0.576,1.045] | 0.095 |

pRBC: packed red blood cell, FFP: fresh frozen plasma, ABT: autologous blood transfusion, PreSpO2: preoperative oxygen saturation, STS: society of thoracic surgeons, ASD: atrial septal defect.
